# 

**Published:** 1910-06

**Authors:** 


*The following donations have been received since the publication of the List in March.*

*May* 31*st*, 1910.

| | | |
|---|---|---|
| American Urological Association (1) .. .. | 1 volume. |
| J. Dacre .. .. .. .. .. .. | 9 volumes. |
| R. Shingleton Smith, M.D. (2) .. .. .. | 2 ,, |

### SEVENTY-SIXTH LIST OF BOOKS.

The titles of books mentioned in previous lists are not repeated.

The figures in brackets refer to the figures after the names of the donors, and show by whom the volumes were presented. The books to which no such figures are attached have either been bought from the Library Fund, or received through the *Journal*.

| | | |
|---|---|---|
| **Achorn, J. W.** .. | *Nature's Help to Happiness* .. .. .. .. | [N.D.] |
| **Ash, E.** .. .. .. .. | *Mind and Health* .. .. .. .. .. .. .. | 1910 |
| **Bardswell, N. D.** .. | *The Expectation of Life after Sanatorium Treatment* .. .. .. .. .. .. .. .. | 1910 |
| **Blair, C.**.. .. .. | *Errors of Refraction* .. .. .. .. .. 2nd Ed. | 1910 |
| **Castellani and A. J. Chalmers, A.** | *Manual of Tropical Medicine* .. | 1910 |
| **Chalmers, A. Castellani and A. J.** | *Manual of Tropical Medicine* .. | 1910 |
| **" D., M."** .. .. | *Ideal Health* .. .. .. .. .. .. 2nd Ed. | 1909 |
| **Daniels and E. Wilkinson, C. W.** | *Tropical Medicine and Hygiene* Pt. I | 1909 |
| **Degrais, L. Wickham and** [—] | *Radiumtherapy* (Trans. by S. E. Dore) .. .. .. .. .. .. .. .. | 1910 |
| **Emerson, C. P.** .. | *Clinical Diagnosis* .. .. .. .. 2nd Ed. | [1908] |
| **Gordon, W.** .. .. | *Rainy Winds and Phthisis Prevalence* .. .. | 1910 |
| **Hewer, Mrs. J. L.** | *Our Baby* .. .. .. .. .. .. .. 12th Ed. | 1910 |
| **Hill, A.** .. .. .. | *The Body at Work* .. .. .. .. .. .. .. | 1908 |
| **Hospital Nurse, A**.. | *" Memories "* .. .. .. .. .. .. .. .. | 1910 |
| **Leftwich, R. W.** .. | *An Index of Symptoms* .. .. .. 4th Ed. | 1910 |
| **Logan, T.** .. .. | *Vetera et Nova* (Ed. by Q. McLennan and P. H. Aitken) .. .. .. .. .. 3 vols. | 1910 |
| **Macfie, R. C.** .. | *Air and Health* .. .. .. .. .. .. .. (2) | [1909] |
| **Mummery, P. L.** .. | *Diseases of the Colon* .. .. .. .. .. .. .. | 1910 |
| **Schnyder, L.** .. | *Alcohol and Alpinism* (Trans. by E. G. Richards) .. .. .. .. .. .. .. .. | 1910 |
| **Schofield, A. T.** .. | *Nervousness* .. .. .. .. .. .. .. .. .. | 1910 |
| **Sinclair, Sir W. J.** | *Semmelweis: his Life and Doctrine* .. .. | 1909 |
| **Stewart and W. A. Turner, T. G.** | *A Text-book of Nervous Diseases* | 1910 |

**Thomson, H. H.** .. *Consumption: its Prevention and Home Treatment* .. .. .. .. .. .. .. .. 1910
**Turner and T. G. Stewart, W. A.** *A Text-book of Nervous Diseases* 1910
**Tweedy and G. T. Wrench, E. H.** *Practical Obstetrics* 2nd Ed. 1910
**Vincent, R.** .. .. *The Nutrition of the Infant* .. .. 3rd Ed. 1910
**White, W. H.** .. *Materia Medica* .. .. .. .. .. 11th Ed. 1909
**Wickham and [—] Degrais, L.** *Radiumtherapy.* (Tr. by S. E. Dore) .. .. .. .. .. .. .. .. .. 1910
**Wilkinson, C. W. Daniels and E.** *Tropical Medicine and Hygiene* Pt. I 1909
**Wrench and E. H. Tweedy, G. T.** *Practical Obstetrics.* .. 2nd Ed. 1910
**Younger, E. G.** .. *Insanity in Every-day Practice* .. 2nd Ed. 1910

### TRANSACTIONS, REPORTS, JOURNALS, &c.

American Association of Obstetricians and Gynæcologists Vol. XXI 1909
American Journal of Obstetrics, The .. .. .. .. .. .. Vol. LX 1909
American Journal of the Medical Sciences, The .. Vol. CXXXVIII 1909
American Medicine .. .. .. .. .. .. .. .. .. .. N.S., Vol. IV 1909
American Pediatric Society, Transactions of the .. .. Vol. XXI 1910
American Urological Association, Transactions of the (1) Vol. III 1909
Archives of Pediatrics .. .. .. .. .. .. .. .. .. Vol. XXVI 1909
Beiträge zur klinischen Chirurgie .. .. .. .. .. .. Bd. LXIV 1909
Boston Medical and Surgical Journal, The .. .. .. Vol. CLXI 1909
British Journal of Children's Diseases, The .. .. .. .. Vol. VI 1909
British Journal of Dermatology, The .. .. .. .. Vol. XXI 1909
British Journal of Tuberculosis, The .. .. .. .. .. Vol. III 1909
Bulletin de l'Académie de Médecine .. .. .. Bds. LXI, LXII 1909
Catalogue of Books for 1909, The English .. .. .. .. .. .. .. 1910
Deutsche Zeitschrift für Chirurgie .. .. .. Bds. XCIX, CI 1909
Department of Neurology, Harvard Medical School, Contributions from .. .. .. .. .. .. .. .. .. .. .. .. .. Vol. IV 1910
Edinburgh Medical Journal .. .. .. .. .. .. .. N.S., Vol. III 1909
Glasgow Medical Journal, The .. .. .. .. .. .. Vol. LXXII 1909
Hospital, The .. .. .. .. .. .. .. .. .. .. .. Vol. XLVI 1909
Hospital—
St. Bartholomew's Hospital Reports .. .. .. Vol. XLV 1910
Indian Medical Gazette, The .. .. .. .. .. .. .. .. Vol. XLIV 1909
Johns Hopkins Hospital Bulletin, The .. .. .. .. Vol. XX 1909
Journal of Experimental Medicine, The .. .. .. .. Vol. XI 1909
Journal of Laryngology, The .. .. .. .. .. .. .. .. Vol. XXIV 1909
Journal of Mental Science, The .. .. .. .. .. .. Vol. LV 1909
Journal of Nervous and Mental Disease, The .. Vol. XXXVI 1909
Journal of Obstetrics and Gynæcology .. .. .. Vol. XVI 1909
Journal of Tropical Medicine, The .. .. .. .. .. .. .. Vol. XII 1909
Journal of the Royal Sanitary Institute .. .. .. Vol. XXX 1909
Medical Annual, The .. .. .. .. .. .. .. .. .. .. .. 1910
Medical Chronicle, The .. .. .. .. .. 4th Ser., Vol. XVII 1909
Medical Record .. .. .. .. .. .. .. .. .. .. Vol. LXXVI 1909

| | | |
|---|---|---|
| Medical Review, The .. .. .. .. .. .. .. .. | Vol. XII | 1909 |
| Medical Society's Transactions .. .. .. .. .. .. | Vol. XXXII | 1909 |
| Münchener medizinische Wochenschrift .. .. .. | Bd. II für | 1909 |
| New York Obstetrical Society, Transactions of the .. .. .. .. | | 1909 |
| Nordiskt medicinskt Arkiv .. .. .. .. .. .. .. .. .. .. .. .. | | 1908 |
| Practitioner, The .. .. .. .. .. .. .. .. | Vol. LXXXIII | 1909 |
| Prescriber, The .. .. .. .. .. .. .. .. .. .. .. | Vol. III | 1909 |
| Progrès médical, Le .. .. .. .. .. .. .. .. .. .. .. .. | | 1909 |
| Quarterly Journal of Medicine, The .. .. .. .. .. | Vol. II | 1908-9 |
| Revue de Thérapeutique médico-chirurgicale .. .. .. .. .. .. | | 1909 |
| Revue hebdomadaire de Laryngologie .. .. .. | Tom. XXIX | 1909 |
| Smithsonian Report for 1908 .. .. .. .. .. .. .. .. .. .. | | 1909 |
| Surgeon-General of the Public Health and Marine Hospital Service of the United States—Annual Report of the .. .. .. .. | | 1909 |
| Public Health Reports of | Vol. XXIV, Pt. 1 | 1909 |
| Surgery, Gynecology and Obstetrics .. | Vols. VI, 1908; IX | 1909 |
| Therapeutic Gazette, The .. .. .. .. | Vol. XXXIII | 1909 |
| Travaux pratiques d'Obstétrique et de Gynécologie .. .. .. .. | | 1908 |
| Verhandlungen der deutschen Gesellschaft für Urologie .. .. .. | | 1909 |
| West London Medical Journal .. .. .. .. .. .. | Vol. XIV | 1909 |
| Willing's Press Guide .. .. .. .. .. .. .. .. .. .. .. .. .. .. | | 1910 |
| Zeitschrift für Urologie .. .. .. .. .. .. .. .. .. .. | Bd. III | 1909 |
| Zentralblatt für Chirurgie .. .. .. .. .. .. .. .. .. .. .. .. .. | | 1909 |
| Zentralblatt für Gynäkologie .. .. .. .. .. .. .. .. .. .. .. | | 1909 |
| Zentralblatt für innere Medicin .. .. .. .. .. .. .. .. .. .. .. | | 1909 |

### MEETINGS OF SOCIETIES.

### Bristol Medico-Chirurgical Society.

### Local Medical Notes.

### BRISTOL.

**University of Bristol.**—Faculty of Medicine :—

EXAMINATION SUCCESSES.

F.R.C.S. Eng.—*Final Examination:* C. A. Joll, M.B., B.S. Lond.; A. J. Wright, M.B., B.S. Lond.

University of London.—*Second Examination for Medical Degrees, Part I:* H. J. Orr-Ewing, J. R. Harris, C. C. Harrison, E. S. Sowerby, W. E. Taylor, G. N. Welsford. *Intermediate Examination:* Kathleen M. Cole, W. Salisbury, G. S. Applegate, (*except Pharmacology*) E. W. Wade. *Final Examination for M.B., B.S.:* A. E. Iles and P. J. Veale.

Conjoint Board of London.—*Elementary Biology:* Louisa M. Lister, P. de S. Smith. *Anatomy and Physiology:* C. Kingston, H. B. Logan. *Medicine:* G. H. Griffiths, A. J. Wigmore, W. A. Reynolds, R. C. Clarke. *Surgery:* H. R. B. Hull,* R. S. S. Statham,* W. T. Clarke.* *Midwifery:* S. Marle, W. W. Pratt.

L.D.S. Eng.—*Mechanical Dentistry and Dental Metallurgy:* R. H. Basker, P. W. Flook, J. W. Gilbert, E. J. Handford, B. L. Morgan, C. H. Wilson. *Metallurgy only:* P. L. Ealand. *Final Examination, Part I:* R. A. Slade, A. H. S. Palmer, B. W. Tyson, F. C. Willows. *Part II:* P. J. Burton,* F. J. Hayman.*

* Denotes qualification.

APPOINTMENTS.

**Bristol Royal Infirmary.**—*Honorary Dental Surgeon:* F. C. Nichols, M.R.C.S., L.R.C.P. Lond., L.D.S. Eng. *Honorary Dental Anæsthetist:* C. H. Osmond, L.F.P.S. Glas. *Resident Obstetric Officer:* R. E. Thomas, M.B., B.S. Lond. *House Surgeon:* T. S. Hele, M.B., B.C. Cantab. *House Physicians:* A. R. Jordan, M.B., B.C. Cantab., and G. B. Harland, M.B., B.S. Lond. *Resident to the Ear, Nose and Throat Department:* S. H. Kingston, M.R.C.S., L.R.C.P. *Casualty Officer:* R. W. Greatorex, M B., Ch.B. Edin.

**Bristol General Hospital.**—The following appointments have recently been made:—*House Physician:* W. T. Quinlan, M.R.C.S., L.R.C.P. *House Surgeon:* D. Robertson, M.B., B.Ch.Edin. *Casualty House Surgeon:* S. E. Malherbe, M.B., B.Ch.Edin. *Assistant House Physician:* A. B. Barton, M.B., B.Ch.Edin.

**Bristol Dispensary.**—T. B. Dixon, M.R.C.S., L.R.C.P., and C. E. K. Herapath, M.B., B.S. Lond., have been appointed Medical Officers.

**Clifton Dispensary.**—H. K. Salisbury, M.R.C.S., L.R.C.P., has been appointed Medical Officer.

**Clifton College.**—J. M. Fortescue-Brickdale, M.A., M.D. Oxon., has been appointed Physician.

**Bradford Royal Infirmary.**—F. C. Morgan, M.R.C.S., L.R.C.P., has been appointed House Surgeon.

**Bristol Royal Infirmary.**—The 174th Annual Report, being that for the year 1909, forms a handsome and well-illustrated volume of 116 pages, and gives a full record of excellent work done in the various departments, and outlines the scheme for new buildings, embracing new surgical wards, new casualty department, and operating rooms, to be connected with the old building by spacious subways. The in-patients numbered 3,819, and the large number of 50,195 out-patients is registered. An increased subscription list is urgently needed.

**General Hospital.**—At the half-yearly meeting of the Governors held on March 14th, presided over by the Lord Mayor, the report presented showed that the Hospital had done more work during the past year than during any time previously; the in-patients numbered 2,700, and the out-patients 39,522, with 5,867 dental out-patients. There were 344 maternity cases. The ordinary income amounted to £10,905, in addition to which there were legacies amounting to £4,482, and special donations towards debt reduction totalling £2,500. The total expenditure was £14,529, a reduction of £450 on 1908. The amount due to the Treasurer now stands at £2,937.

**Royal Hospital for Sick Children and Women.**—At the annual meeting held recently the report showed that the expenditure exceeded the income during 1909 by £160, the income being £4,559, and the expenditure £4,719. The in-patients numbered 754 children and 60 women, and 3,512 children and 1,491 women had attended as out-patients. The Weston-super-Mare Sanatorium had received 65 convalescent patients.

### UNIVERSITY OF BRISTOL.

**Tropical Medicine.**—A series of lectures on Tropical Medicine was recently delivered in the Medical Faculty by Dr. A. J. M. Bentley. It would be well to persevere in this line of work, as Bristol offers many advantages as a centre for the study of tropical diseases.

**University Settlement.**—A committee has been formed to consider the formation of a University Settlement, the aims of which would be: (*a*) to promote the general welfare of the neighbourhood in which it is situated, (*b*) to provide a centre for the systematic study of social and industrial conditions. The committee considers that the Settlement should include a residence for women, with library and lecture and club rooms for both men and women workers. The importance of training experts in social work is pointed out, and the sympathy of students asked for. About £1,000 would be required for an initial outlay, and the annual cost of maintenance would be about £300. Already £220 has been promised towards the initial cost, although no appeal has as yet been issued.

**Royal College of Surgeons of England, Honorary Medal.**—As will be seen from the report of the last meeting of the Council of the Royal College of Surgeons, the Honorary Medal of the College was awarded to Dr. Robert Fletcher, M.R.C.S., of Washington, U.S.A. Dr. Fletcher has rendered conspicuous service to medical literature. In 1876 he began to assist in the charge of the library in the office of the Surgeon-General of the United States Army. He afterwards became associate editor, and subsequently editor-in-chief of that unique work, the *Index-Catalogue of the Library of the Surgeon-General's Office*. He was also editor, at first in association with Dr. Billings and since alone, of that useful publication, the *Index-Medicus*. We are glad to claim Dr. Fletcher as a fellow-countryman. He was born at Bristol eighty-four years ago, and began his medical education at the Bristol Infirmary, completing it, we believe, at the London Hospital. He was admitted a Member of the Royal College of Surgeons of England in 1844. Going to the United States, he practised for some time in Cincinnati. He served with credit during the Civil War, being commissioned as

Surgeon of the United States Volunteers and breveted Colonel. We heartily congratulate Dr. Fletcher on the distinction which has been conferred upon him; it is one of the highest the Royal College of Surgeons has it in its power to bestow. The Honorary Medal was instituted in 1802. It is a gold medal having on the obverse the armorial bearings, crest, supporters and motto of the College, and on the reverse Galen contemplating a human skeleton. The leading considerations in awarding the medal are "liberal acts or distinguished labours, researches and discoveries eminently conducive to the improvement of natural knowledge and of the healing art." It has been awarded only ten times. In the list of recipients are the names of Sir Richard Owen (1883), Sir Erasmus Wilson (1884), Sir James Paget (1897), Lord Lister (1897), and Sir Richard Havelock Charles (1906).—*Brit. M. J.*, 1910, vol. i, p. 1015.

**Winsley Sanatorium.**—Referring again to the annual report of this Institution (which was commented on in our last issue), it will be noted that during 1909 the number of beds available was 68. Of these 43 are maintained, and 9 leased to various authorities. Of the remaining 16, 12 are reserved for patients willing to pay 15s. per week, and 4 are utilised by the committee for patients who pay 35s. to 42s. per week. The medical report shows that 248 were admitted during 1909, and 236 discharged. Of these 111 left fit for work, 82 improved, 37 not improved, and 6 died in the Sanatorium. The capital debt now amounts to £9,000.

Report of Medical Board.—The usual meetings have been held in accordance with the rules, when the medical visitors have been appointed, applications from doubtful cases have been considered, and the questions referred to the Board have been dealt with. There are now forty-three names on the list: they are, so far as possible, those of physicians who are doing medical work only, but clearly this is not possible in the country districts.

The medical attendant of the patient who signs the first report, which is presumably on behalf of the patient, is at liberty to send his patient to anyone whose name is on the list for the second certificate, which is presumably on behalf of the Sanatorium, and by one who knows, or is expected to know, what cases are desirable in accordance with the objects and aims of the Sanatorium authorities. It would be an excellent arrangement if all cases could be examined by the medical officer to the institution before admission, but it is clearly not possible that all cases shall take a journey to the Sanatorium for this purpose, and it is equally impossible that our medical officer shall spend his time in travelling about the three counties. The time of the resident medical superintendent is fully occupied in the work of the Sanatorium itself; he has quite the maximum number of cases to supervise daily, but, nevertheless, he is at all times willing to fill in the second certificate on behalf of the Board, of which he is a member.

In endeavouring to select the most suitable cases for Sanatorium treatment, much has been expected from the newer methods of diagnosis, and many tests have been devised which are more or less reliable; in doubtful cases these tests should be of great assistance, but often it happens that they cannot be applied until the patient has already been accepted as a Sanatorium patient.

At the Tuberculosis Conference, held last year at Oxford, much discussion on tuberculosis in children took place; it was pointed out that pulmonary tuberculosis pure and simple was essentially a disease of adult life, whilst children often succumbed to meningeal, abdominal, or more often to a generalised tuberculosis. It has often been found that at the end of the first year some 8 per cent. of all infants dying were found to be tuberculous, and of all children submitted to the *post-mortem* examination under fifteen years of age tuberculosis was found in something like 40 per cent. of the cases; nevertheless it is well known that these cases of children are particularly amenable to treatment, and hence the wisdom of Mr. Arkell's action in endowing a bed for the children of Wiltshire. Unfortunately the age limit laid down by our rules (ten years) must sometimes detract from the utility of such a bed, but it is hoped that cases occurring between the ages of ten and twenty will be given the advantage of Sanatorium life as much as possible, as it must be at this period of life that the success of treatment is most assured and most lasting. It is interesting to note that several of our cases of phthisis in children have been discovered in consequence of the systematic medical inspection of schools.

We trust that the idea that a Sanatorium is of any danger or disadvantage to the neighbourhood in which it is situated is no longer entertained. Medical men know well that the safest place foı those with a predisposition to tubercle is a well conducted Sanatorium, and that it is only the ignorant and careless subject of advanced tuberculosis who is a danger to the community, and should be carefully guarded; the tubercle bacillus is now so much under control that we need hear no more of the bacillus scare.

